# Understanding prescribed dose in hand strengthening exercise for rheumatoid arthritis: A secondary analysis of the SARAH trial

**DOI:** 10.1002/msc.1646

**Published:** 2022-05-16

**Authors:** Graham Boniface, Maria T. Sanchez‐Santos, Meriel Norris, Neil OConnell, Esther Williamson, Sarah E. Lamb

**Affiliations:** ^1^ Department of Health Sciences Centre for Health and Wellbeing across the Lifecourse Brunel University London Uxbridge UK; ^2^ Nuffield Department of Orthopaedics Rheumatology and Musculoskeletal Sciences (NDORMS) University of Oxford Oxford UK; ^3^ College of Medicine and Health University of Exeter Exeter UK

**Keywords:** dose, hand, hand exercise, hand strength, rheumatoid arthritis, SARAH trial

## Abstract

**Objective:**

1) To identify therapist or participant characteristics associated with prescribed dose of hand strengthening exercise in adults with rheumatoid arthritis and 2) To determine the impact of dose prescribed on outcome (hand function and grip strength).

**Methods:**

Overall dose was calculated using area under the curve (AUC). Analysis 1 assessed the association between therapist professional background, therapist grade, baseline participant physical and psychological characteristics and prescribed dose. Analyses 2 and 3 estimated the relationship between prescribed dose and overall hand function and grip strength. Generalised estimating equation linear regression analysis was used.

**Results:**

Analysis 1: Being treated by an occupational therapist (*β* = −297.0, 95% CI −398.6, −195.4), metacarpophalangeal joint deformity (*β* = −24.1, 95% CI −42.3, −5.9), a higher number of swollen wrist/hand joints (*β* = −11.4, 95% CI −21.6, −1.2) and the participant feeling downhearted and low all of the time (*β* = −293.6, 95% CI −436.1, −151.1) were associated with being prescribed a lower dose. Being treated by a grade 6 therapist (*β* = 159.1, 95% CI 65.7, 252.5), higher baseline grip strength (*β* = 0.15, 95% CI 0.02, 0.28) and greater participant confidence to exercise without fear of making symptoms worse (*β* = 18.9, 95% CI 1.5, 36.3) were associated with being prescribed a higher dose. Analyses 2 and 3: Higher dose was associated with greater overall hand function (*β* = 0.005, 95% CI 0.001, 0.010) and full‐hand grip strength (*β* = 0.014, 95% CI 0.000, 0.025) at 4‐month.

**Conclusion:**

Higher dose was associated with better clinical outcomes. Prescription of hand strengthening exercise is associated with both therapist and participant characteristics.

## 1 INTRODUCTION

Hand exercise is recommended by the National Institute for Health and Care Excellence (NICE) for individuals with pain and dysfunction of the hands and wrists caused by rheumatoid arthritis (NICE, 2018). This recommendation is underpinned by data from the strengthening and stretching for rheumatoid arthritis of the hand (SARAH) trial, which found that providing a tailored hand exercise programme in addition to usual care, is a clinically and cost‐effective adjunct to the various drug regimens presently recommended (Lamb et al., 2015). Post‐hoc analysis of the SARAH programme identified the techniques used to target grip strength (i.e. hand strengthening exercise) partially mediated improvements in overall hand function (Hall et al., 2017). Whilst NICE support using strengthening exercise for the rheumatoid hand, no information is provided about what dose may work best.

Developing a better understanding of the optimal dose for rehabilitation interventions has recently been identified as a research priority (CSP, 2018a). Further refinement of the dose of hand strengthening exercise may optimise clinical and cost‐effectiveness and inform future (more detailed) clinical guidelines. Such refinement should be informed by data and evidence as far as possible. We used data from the SARAH trial to address the following objectives:

## 2 OBJECTIVES

To identify the therapist and participant characteristics at baseline associated with the overall dose of hand strengthening exercise prescribed to participants in the SARAH trial (Analysis 1).

To identify the association between overall dose of hand strengthening exercise prescribed during the programme and hand function and grip strength (Analyses 2 and 3).

## 3 METHODS

### 3.1 Design

This study is a post‐hoc exploratory analysis of the data from the SARAH trial. The SARAH trial was a pragmatic parallel‐group trial conducted at 17 National Health Service sites across the UK. The complete methods of the SARAH trial are described in full elsewhere (Williams et al., 2015).

#### 3.1.1 SARAH hand exercise programme

The programme comprised six sessions of face‐to‐face contact (one assessment and five supervised exercise sessions) with an occupational therapist or physiotherapist. Seven mobility and four strengthening exercises were used. The four strengthening exercises (eccentric wrist extension, gross grip, pinch grip, finger adduction) used load (resistance) provided by bands, balls or therapeutic putty. Therapists followed a predefined protocol for prescribing the dose (sets, repetitions and load) of each strengthening exercise. Intensity was set using the Borg rating of perceived exertion scale (Borg, 1982) and each exercise was progressed or regressed according to both participant capability and therapist judgement. The goal was for the participant to perform each exercise, where possible at a volume and load that was achievable while still providing a stimulus for physiological change (Heine et al., 2012).

### 3.2 Data collection

We used data provided by participants at baseline and 4‐month follow‐up. Data describing the prescribed hand strengthening exercise was extracted from the exercise treatment logs that were completed by therapists at each exercise session (Williams et al., 2015), including dose parameters (sets, repetitions and load). Where exercise treatment logs contained insufficient/ambiguous information about dose, we utilised the personal exercise diaries completed by both the therapist and participant to assist with completion.

### 3.3 Participants

Between 05/10/2009 and 10/05/2011, 490 participants with a rheumatoid arthritis (RA) diagnosis according to the American College of Rheumatology clinical and immunological criteria (American College of Rheumatology Subcommittee on Rheumatoid Arthritis, 2002), with pain and dysfunction of the hands and/or wrist joints, who were not on a disease‐modifying antirheumatic drugs (DMARD) regime, or had been stable on a DMARD regime (including biological agents if used) for 3 months or more were recruited. 244 participants were randomly assigned to usual care and 246 to the tailored exercise programme. Usual care included information published by Arthritis Research UK, joint‐protection education and, where indicated, functional splinting.

### 3.4 Ethical approval

Ethical approval for this study was granted by Brunel University London Research Ethics Committee (13763‐LR‐Jan/2019‐ 17,357‐1). Original ethical approval for the SARAH trial (ISRCTN registration number: 89,936,343) was gained from the Oxford **C** Multi‐Centre Research Ethics Committee (MREC 08/H0606/47).

### 3.5 Prescribed dose of hand strengthening exercise

The overall dose of hand strengthening exercise that participants were prescribed at the five supervised exercise sessions was calculated using the area under the curve (AUC) method. This approach has previously been used for identifying response to an intervention and considers the change in the value of a parameter over time (Matthews et al., 1990; Pruessner et al., 2003). We focussed exclusively on the dose prescribed and completed at the five supervised exercise sessions due to well recognised problems with recording exercise adherence to home exercise (Nicolson et al., 2018). Table 1 provides a guide for a participant attending all five supervised exercise sessions. For example, if the participant was prescribed 1 × 10 repetitions using yellow band, ball or therapeutic putty for each of the four strengthening exercises used in the SARAH programme, the prescribed overall dose would be 640 AUC. A detailed description of the approach is provided (see Additional file 1).

**TABLE 1:** A guide to interpreting dose calculated using AUC

Volume	Load (resistance used)^a^							
Sets x Repetitions	Nil load	White	Tan	Yellow	Red	Green	Blue	Black
**1 x 10**	160	320	440	640	800	960	1120	1280
**2 x 10**	320	640	880	1280	1600	1920	2240	2560
**3 x 10**	480	960	1320	1920	2400	2880	3360	3840

^a^ White = Lowest resistance, Black = Highest resistance of band, ball or therapeutic putty.

### 3.6 Candidate predictors for prescribed dose

Based on theoretical knowledge and the clinical experience of physiotherapists within the research team, we selected candidate predictors potentially associated with the overall dose prescribed (Table 2).

**TABLE 2:** Selected candidate predictors of overall dose prescribed

Candidate predictors	How measured
Age	Measured in years
Gender	Female
	Male
Therapist profession	Occupational therapist
	Physiotherapist
Therapist grade (Agenda for Change^a^)	Band 5
	Band 6
	Band 7
Active wrist extension	Measured in degrees by goniometer
Hand and wrist swelling count	0‐22 (0 = No swollen joints)
Hand and wrist tenderness count	0‐22 (0 = No tender joints)
Mean combined finger flexion	Measured in millimetres by ruler
Metacarpophalangeal (MCP) joint deformity	Deformity present (Radial/ulnar)
	No deformity
Thumb opposition^b^	Measured using the Kapandji scale (0‐10, 10 = best opposition)
Full‐hand grip strength	Newtons (measured by dynometer)
Tripod grip strength	Newtons (measured by dynometer)
Confidence to perform exercise without making symptoms worse	0 ‐ 10 (10 = Totally confident)
Michigan hand outcomes questionnaire (MHQ) overall hand function subscale score^c^	0 ‐ 100 (100 = greater function)
Pain frequency	Rarely/never
	Sometimes
	Always/often
Pain severity	Very mild/mild
	Moderate
	Severe/very severe
Short‐Form survey (SF‐12) question^d^ ‐ have you accomplised less than you would like?	A little/most of the time
	Some of the time
	All/most of the time
Short‐Form survey (SF‐12) question^d^ ‐ have you felt downearted and low?	A little/most of the time
	Some of the time
	All/most of the time
Years diagnosed with RA	Measured in years

^a^ (NHS Careers, 2021).

^b^ (Kapandji, 1992).

^c^ (Chung, Pillsbury, Walters, & Hayward, 1998).

^d^ (Jenkinson & Layte, 1997).

### 3.7 Hand function and grip strength

Hand function and grip strength at 4 months follow‐up (closest to the supervised exercise sessions ending) were the outcomes used in our model to evaluate association with exercise dose. MHQ overall hand function was the primary outcome in the SARAH trial, and hand grip strength is known to partially mediate overall hand function (Hall et al., 2017).

## 4 STATISTICAL ANALYSIS

Data was analysed using IBM SPSS Statistics for Windows, version 26.0 (IBM Corp., Armonk, N.Y., USA). The unit of analysis was the hand. Distribution of the outcome and each candidate variable was described using mean and standard deviation, median (interquartile range), as well as tabulations of frequency and percentage.

### 4.1 Analysis 1

#### 4.1.1 Steps

1. Distribution of continuous independent and dependent variables were checked for normality using histograms. Possible errors were also checked within the data.
2. Generalised estimating equation (GEE) univariate analysis: Variables with a (*p* < 0.10) were included from the multivariate analysis. For each participant, an exchangeable correlation matrix was used to adjust for correlation between each hand.
3. Multivariate analysis: To select factors associated with prescribed overall dose, backward stepwise regression was used with a *p* < 0.05 used as a cut off. Only complete cases were used.
4. Generalised estimating equations (GEE) linear regression. Coefficients (B) with 95% confidence intervals (CIs) were calculated.

### 4.2 Analyses 2 & 3

#### 4.2.1 Steps

1. Each predictor variable identified in analysis 1 with a *p* < 0.10 was included in the univariate analysis.
2. GEE Univariate analysis: Along with prescribed overall dose (independent variable), we evaluated whether each predictor variable was associated with the dependent variable of outcome (4‐month overall hand function or full‐hand grip strength). Adjustment was made for baseline overall hand function and full‐hand grip strength respectively. Only complete cases were used.
3. Variables associated with both prescribed overall dose and outcome (p‐value < 0.05) were included in the multivariate analysis.
4. GEE linear regression. Coefficients (B) with 95% confidence intervals (CIs) were calculated.

## 5 RESULTS

### 5.1 Characteristics of participants included

Of the 246 participants randomised to the tailored exercise programme, 24 (9.7%) were excluded: 19 because no hand strengthening exercise was prescribed (e.g., withdrew from treatment) and 5 because their exercise treatment logs were recorded as missing (e.g., unable to calculate dose). Participants who had no hand strengthening exercise prescribed were younger (mean (SD; standard deviation) 56.4 (15.5) versus 61.6 (11.9)), had a longer diagnosis of RA (mean (SD; standard deviation) 14.0 (10.3) versus 12.4 (10.1)) and more frequently (always/often) reported pain (73.7% vs. 61.7%). Participant characteristics are described in more detail (see Additional file 2).

### 5.2 Prescribed overall dose

Overall dose of hand strengthening exercise was calculated for 222/246 (90.2%) participants (Table 3). Of the four exercises, gross grip had the highest overall dose prescribed compared to eccentric wrist extension, finger adduction and finger pinch.

**TABLE 3:** Summary statistics of prescribed overall dose (AUC) (*n* = 222)

Strengthening exercise	Hand side	Median (IQR)
Eccentric wrist extension	Left:	160.0 (80.7, 205.0)
	Right:	160.0 (80.7, 205.0)
Gross grip	Left:	260.0 (195.8, 391.2)
	Right:	260.0 (200.0, 382.5)
Finger adduction	Left:	138.7 (74.6, 195.0)
	Right:	137.5 (75.7, 195.0)
Finger pinch	Left:	162.5 (104.2, 220.0)
	Right:	165.0 (108, 217.0)
Prescribed overall dose	Left:	725.0 (501.8, 1017.0)
	Right:	731.5 (529.3, 1025.0)

### 5.3 Analysis 1 (factors associated with prescribed overall dose)

Following univariate analysis (Table 4), therapist type, therapist grade, MCP joint deformity, thumb opposition, full‐hand grip strength, tripod grip strength, overall hand function, hand and wrist swollen joint count, hand and wrist tender joint count, pain severity, accomplishing less than you would like, feel down hearted or low, confidence to perform exercise without fear of making symptoms worse, age, gender, years since diagnosed with RA were included in the backward stepwise multivariate regression. In this model we found a mix of therapist, participant physical and psychological factors were predictive of the prescribed overall dose. We report these below:

**TABLE 4:** Analysis 1: Univariate analysis of candidate predictors

Predicting factors	No. participants/No. Hands	Coef. (*β*)	95% CI	*p*‐value
Therapist factors:				
Type of therapist (reference category: Physiotherapist)	222/444			
Occupational therapist		−272.0	−378.0; −166.0	0.00^a^
Therapist grade (reference category: job band 7)	213/426			
Job band 5		−129.6	−352.8, 93.4	0.25
Job band 6		97.6	−3.1, 198.4	0.05^a^
Participant physical factors:				
MCP joint deformity (reference category: No deformity)	221/442	−22.1	−39.3; −4.9	0.01^a^
Active wrist extension	222/444	0.1	−0.2, 0.6	0.41
Composite finger flexion	222/443	−0.3	−0.8, 0.1	0.13
Thumb opposition	222/444	2.3	−0.1, 4.7	0.06^a^
Full‐hand grip strength	221/441	0.1	0.03, 0.2	0.01^a^
Tripod grip strength	219/436	0.7	0.3, 1.1	0.00^a^
Overall hand function	222/444	0.3	−0.05, 0.6	0.05^a^
Hand/wrist tender joint count	222/444	−10.2	−18.9, −1.5	0.02^a^
Hand/wrist swollen joint count	222/444	−11.3	−21.8, −0.9	0.03^a^
**Participant reported factors:**				
Age into 4 categories (reference category: 55–64 years old)	222/444			
Less than 45		−171.1	−352.6; 10.4	0.06^a^
45‐54		−86.9	−263.3; 89.5	0.33
65 and over		−80.1	−202.2; 42.0	0.19
Sex (reference category: male)	222/444			
Female		−21.7	−49.8; 6.3	0.12^a^
Years since diagnosed with RA	221/442	−4.9	−9.66, −0.19	0.04^a^
Pain frequency (reference category: Rarely/never)	222/444			
Always/often		−36.2	−186.7, 114.3	0.63
Sometimes		12.2	−161.8, 186.4	0.89
Pain severity (reference category: Very mild/mild)	216/432			
Moderate		−49.7	−169.9; 70.4	0.41
Severe/very severe		−151.1	−292.2; −10.1	0.03^a^
Accomplished less than you would like (reference category: A little/none of the time)	222/444			
All/Most of the time		−209.9	−333.2, −86.5	0.00^a^
Some of the time		−92.8	−224.1, 38.4	0.16
Feeling downhearted or low (reference category: A little/none of the time)	222/444			
All/most of the time		−364.4	−532.0, −196.8	0.00^a^
Some of the time		−86.1	−203.5, 31.8	0.15
Confidence to perform exercise	221/442	21.3	2.7, 39.9	0.02^a^

^a^ Variables with a (*p <* 0.10).

#### 5.3.1 Therapist factors

When the prescribing therapist was an occupational therapist, participants received (*β* = −297.0 AUC, 95% CI −398.6, −195.4, *p* ≤ 0.001) less overall dose compared to when the clinician was a Physiotherapist. Participants were prescribed greater overall dose of strengthening exercise (*β* = +159.1 AUC, 95% CI 65.7, 252.5, *p* ≤ 0.001) when their therapist was a grade 6 compared to when their therapist was a grade 5 or grade 7.

#### 5.3.2 Participant physical factors

Participants with MCP joint deformity (radial or ulnar drift) were prescribed (*β* = −24.1 AUC, 95% CI −42.3, −5.9, *p* ≤ 0.009) less overall dose compared to those participants with no deformity. Swollen joints count was also associated with less overall dose being prescribed. For each swollen joint recorded, overall dose reduced by (*β* = −11.4 AUC, 95% CI −21.6, −1.2, *p* ≤ 0.028). In contrast, for each 1 N increase in full‐hand grip strength recorded at baseline, the prescribed overall dose increased by (*β* = +0.15 AUC, 95% CI 0.02, 0.2, *p* ≤ 0.016).

#### 5.3.3 Participant psychological factors

Participants who reported feeling downhearted or low all of the time were prescribed (*β* = −293.6 AUC 95% CI −436.1, −151.1, *p* ≤ 0.001) less overall dose when compared to those feeling downhearted less often. Conversely, participants who reported a greater confidence to exercise on a scale of 1–10 (10 = most confident) were associated with being prescribed a greater overall dose of hand strengthening exercise (*β* = +18.9 AUC, 95% CI 1.5, 36.3).

### 5.4 Association between prescribed overall dose and outcome

#### 5.4.1 Analysis 2 (overall hand function)

Of the 246 participants, we excluded 29 (11.7%): 24 where the outcome (overall hand function) was missing at baseline and/or 4‐month follow up and 5 because their exercise treatment logs were recorded as missing (e.g., unable to calculate dose). Potential confounders (thumb opposition, full‐hand grip strength, participant age, years diagnosed with RA, therapist grade, pain severity and confidence to perform exercise without making symptoms worse) were included in the multivariate analysis. Higher overall exercise dose was associated with better outcomes in function at 4‐month. For every 1 AUC, overall hand function increased by *β* = 0.005 points (95% CI 0.001, 0.010, *p* = 0.027).

#### 5.4.2 Analysis 3 (full‐hand grip strength)

Of the 246 participants, we excluded 55 (22.3%), 50 where the outcome (full‐hand grip strength) was missing at baseline and/or 4‐month follow up and 5 participants because their exercise treatment logs were recorded as missing (e.g., unable to calculate dose). Potential confounders (thumb opposition, years diagnosed with RA, and therapist type) were included in the multivariate analysis. Higher overall exercise dose was associated with better outcomes in function at 4 months. For every 1 AUC, full‐hand grip strength increased by *β* = 0.014 N (95% CI 0.00, 0.02, *p* = 0.045).

## 6 DISCUSSION

This study provides evidence that being prescribed a higher overall dose of hand strengthening exercise is associated with better clinical outcomes. It indicates that the prescription of hand strengthening exercise is a complex multi‐factorial process, associated with both therapist and participant characteristics. Greater full‐hand grip strength at baseline, having strengthening exercise prescribed by a grade 6 therapist and the participant being more confident to exercise without fear of making symptoms worse was associated with a higher overall dose. Conversely MCP joint deformity, having hand strengthening exercise prescribed by an occupational therapist opposed to a physiotherapist, the participant reporting feeling downhearted all of the time and a higher number of swollen wrist/hand joints was associated with a lower overall dose.

Limited evidence exists to ascertain the most effective dose of hand exercise in RA. Higher intensities of exercise is tentatively recommended over lower intensities (Bergstra et al., 2014; Hammond & Prior, 2016). This study supports clinicians aiming to prescribe higher overall dose with their patients in order to achieve better outcomes. What is less well understood from our results, is whether volume (i.e. sets and repetitions) is more or less important than load (i.e. resistance) used. Exercise‐based clinical trials may better inform the development of future guidelines if more detailed dose‐response information is offered as part of the dissemination process.

Both therapist professional background and job grade (our surrogate for clinical experience) were associated with dose. Clinician professional background, years of experience, knowledge, beliefs, attitudes, and behaviour towards exercise have previously been associated with how exercise is prescribed in the rehabilitation setting (Bennell et al., 2014; Eulenburg et al., 2015; Hansen et al., 2018). Differences in professional training may be one possible reason. Physiotherapy as a profession appears to place a stronger emphasis on movement and exercise (CSP, 2018b). Consequently this profession maybe more confident to progress participants or prescribe higher initial dose of hand exercise. Identifying how therapists select, weight and combine information when deciding what dose of hand exercise to prescribe may be helpful in reducing variation in prescribing practice and optimising clinical and cost effectiveness.

Two indices of disease activity (MCP joint deformity and joint swelling) were associated with dose. MCP joint deformity has been reported as a reliable indicator of impaired hand function and grip strength in RA (Dias et al., 2012); Vliet Vlieland et al. (1996). Joint swelling is commonly associated clinical feature with RA (NHS, 2019). Swelling has been proposed to influence the range of joint movement and grip strength (Fraser et al., 1999; Scott & Houssien, 1996). Participants with greater grip strength measured at baseline had a higher dose. Previous research suggests grip strength has been identified as an important marker for hand function (Higgins et al., 2018). Two participant psychological factors (mood and confidence) were found to be associated with dose. Exercise and its positive effects on mood are well known (Cooney et al., 2013). Less understood are the effects of mood on dose prescribed in exercise‐based clinical trials. Depression is considerably higher amongst individuals with RA (Katon & Schulberg, 1992). Participant reporting of feeling downhearted or low all of the time was associated with lower dose being prescribed. Higher participant confidence to exercise without fear of making their symptoms worse was associated with higher prescribed dose. In a qualitative study that interviewed SARAH trial participants, confidence was identified as a facilitator for performing and adhering to the exercises (Nichols et al., 2017). Those participants with lower confidence levels may need more support to engage and progress the exercises. Evaluating these factors may help therapists to work with participants to achieve greater doses of hand strengthening exercise.

## 7 STUDY STRENGTHS AND LIMITATIONS

This study utilised a relatively large trial data set and our analyses have controlled for a range of variables relating to baseline function, condition severity and participant characteristics. We recognise our study has some important limitations. These findings are based on observational data from within an RCT and our analyses were not pre‐planned as part of that trial. Whilst we believe the overall dose calculated for the five supervised sessions acts as a reasonable proxy for dose completed over the 12 week programme, it may not fully reflect changes in the participants ability to perform the strengthening exercises (for example during symptom flare‐up, injury or illness). We assigned a numerical rating to each level of resistance to help with calculating dose. This was because we were unable to obtain information on resistance level for exercise balls and putty (i.e. colour equating to kg). We accept this approach may have influenced the overall dose calculated. There may also have been other factors influencing prescribed dose (e.g. therapists knowledge/training, beliefs or access to equipment such as exercise band/putty). Considering some of the difficulties in calculating overall dose, future trials using strength‐based exercise should explicitly report load/resistance in metric terms (e.g. kilograms).

## 8 CONCLUSION

Clinicians using the SARAH hand programme with their patients should aim to prescribe higher overall dose of strengthening exercise if they wish to help patients achieve greater overall hand function and grip strength. Further research into understanding about how therapists select, weight and combine information gathered during the healthcare consultation when prescribing dose may be useful for informing future clinical practice.

## CONFLICTS OF INTEREST

None.

## ETHICS STATEMENT

Ethical approval for the current project was granted by Brunel University London Research Ethics Committee (13763‐LR‐Jan/2019‐ 17,357‐1). The SARAH trial (ISRCTN registration number: 89,936,343) was originally approved by the Oxford C Multi‐Centre Research Ethics Committee (MREC 08/H0606/47) in addition to the research and development departments for each NHS site.

## AUTHOR CONTRIBUTION

Graham Boniface devised the study, collected the data, performed the analysis and wrote the manuscript. Maria T. Sanchez‐Santos supported Graham Boniface with statistical analysis. Maria T. Sanchez‐Santos, Meriel Norris, Neil OConnell, Esther Williamson, Sarah E. Lamb supported Graham Boniface during the study and reviewed the manuscript.

## Supporting information

Supplementary Material

Supplementary Material

## Data Availability

Research data are not shared.
