# Supplementary material for: Understanding prescribed dose in hand strengthening exercise for rheumatoid arthritis: A secondary analysis of the SARAH trial
Source: Musculoskeletal Care. 2022 May 16;20(4):899–907. doi: 10.1002/msc.1646 (PMC10084296; doi:10.1002/msc.1646)
Supplement: Supplementary file 1 — Supplementary Material [file MSC-20-899-s001.docx]

**APPENDIX A**

**Calculating overall dose of strengthening exercise:**

**Overview:**

In exercise-based clinical trials, clear and accurate reporting of dose parameters (e.g. sets, repetitions, load) have been identified as important for helping practitioners replicate interventions in clinical practice (Slade, Dionne, Underwood, & Buchbinder, 2016; Slade, Dionne, Underwood, Buchbinder, et al., 2016). In exercise-based clinical trials in RA, these parameters in relation to strengthening exercise are often incomplete. In particular, the load (kg/lbs) used by trial participants is poorly reported and when it is, it is commonly reported as a % of a participant’s one-repetition maximum (1RM) (Boniface et al., 2020). This makes analysing dose-response problematic. In the SARAH trial the dose of strengthening exercise that participants were exposed to at the five supervised exercise sessions was recorded using the following dose parameters: sets, repetitions and load. Load was provided by therabands, therapy putty or hand exerciser balls. Load was denoted by the colour of the equipment used where it is common for colour to denote the level of resistance (e.g. red = medium resistance). Owing to the lack of consistent information about load (kg/lbs) related to these types of equipment, either in the literature or from the manufacturers. we took a pragmatic decision and assigned a load value for each different colour.

To calculate overall dose of strengthening exercise, we first calculated the dose of strengthening exercise for each individual exercise (wrist extension, gross-grip, finger adduction and pinch-grip) for both left and right hands. We opted to find the area under the curve (AUC), a central task in calculus, using Microsoft Excel. To calculate AUC, we theoretically mapped (e.g. didn’t physically draw the graph) the trendline for dose of strengthening exercise and then determined the four integrals underneath the trendline. AUC as a methodological approach has previously been used for identifying a response to an intervention and takes into account the change in value of a parameter (i.e. overall dose of strengthening exericse) over time (Matthews, Altman, Campbell, & Royston, 1990; Pruessner, Kirschbaum, Meinlschmid, & Hellhammer, 2003). We will use a participant from the SARAH trial as an example to describe the approach:

**Example:**

The following example is intended to aid the reader in understanding how we calculated the overall cumulative dose of strengthening exercise for each participant. We present a step by step worked example below using one of the actual participants allocated to receive the SARAH exercise programme.

**Participant 5**

Participant 5 attended four out of the five supervised exercise sessions with a SARAH trained therapist. They missed one exercise session (session 4) due to being unwell. Each of the four strengthening exercises (wrist extension, gross-grip, finger adduction and pinch-grip) used had the following dose parameters (sets, repetitions and load) recorded in the exercise treatment logs (Table 1) by the SARAH therapist. This represents the therapist prescribed dose of strengthening exercise completed by the participant at the face-to-face session across all four exercises.

**Table 1.** Participant ‘5’ documented strength exercise data

| Exercise session | Eccentric wrist extension | | | Gross grip | | | Finger adduction | | | Finger pinch | | |
| --- | --- | --- | --- | --- | --- | --- | --- | --- | --- | --- | --- | --- |
|  | Sets | Reps | Load | Sets | Reps | Load | Sets | Reps | Load | Sets | Reps | Load |
| 1 | 1 | 10 | Red | 1 | 10 | Yellow | 1 | 10 | Yellow | 1 | 10 | Yellow |
| 2 | 1 | 10 | Red | 1 | 10 | Yellow | 1 | 10 | Yellow | 1 | 10 | Yellow |
| 3 | 1 | 10 | Red | 1 | 10 | Red | 1 | 10 | Yellow | 1 | 10 | Yellow |
| 4 | - | - | - | - | - | - | - | - | - | - | - | - |
| 5 | 1 | 10 | Red | 1 | 10 | Yellow | 1 | 10 | Yellow | 1 | 10 | Yellow |

To calculate the overall cumulative dose prescribed by the therapist and completed by the participant we completed the following steps in Microsoft Excel.

**Step 1:**

For each exercise across the five exercise session, we completed the following three processes:

1) We multiplied sets by repetitions to calculate the volume (e.g. 1x10=10).

2) We replaced the load (denoted by colour) with a corresponding load value (Table 2) (e.g. red=5).

3) We multiplied volume by the load value to calculate dose (Table 3). Graphically, dose for each exercise across the five exercise sessions is shown below (Figure 1)

**Table 2.** Corresponding load value

| Resistance colour | Load value | Resistance colour | Load value |
| --- | --- | --- | --- |
| Nil load used | 1 | Green | 6 |
| White | 2 | Blue | 7 |
| Cream/Flesh/Pink/Tan | 3 | Black | 8 |
| Yellow | 4 | Silver^†^ | 9 |
| Red | 5 | Gold^†^ | 10 |

^†^ Silver and gold are colours used to denote the strongest TheraBands

**Table 3.** Participant ‘5’ strength exercise data

| Exercise session | Eccentric wrist extension | Gross grip | Finger adduction | Finger pinch |
| --- | --- | --- | --- | --- |
|  | Dose | Dose | Dose | Dose |
| 1 | 50 | 40 | 40 | 40 |
| 2 | 50 | 40 | 40 | 40 |
| 3 | 50 | 50 | 40 | 40 |
| 4 | 0 | 0 | 0 | 0 |
| 5 | 50 | 40 | 40 | 40 |

Figure 1. Dose trendlines for each strengthening exercise

**Step 2:**

To calculate overall AUC for each strengthening exercise, we added together the four integral areas (i.e. exercise sessions 1-2, exercise sessions 2-3, exercise sessions 3-4 and exercise sessions 4-5) under the trendlines (Figure 1) using Microsoft Excel (Table 4). Using wrist extension as an example, we used the following formula =SUM(B1+B2)/2*(A2-A1) to calculate the four integral AUC (Column C). For example, to calculate the integral AUC between session 1 and 2, the equation would be: Integral AUC=SUM(50+50)/2*(2-1). We repeated this equation for calculating integrals between exercise sessions 2 and 3, 3 and 4 and 4 and 5. Note there is no value recorded in Column C, Row 5. This is because, in Excel, we delete the value in the cell as it references nothing in what would be Row 6. We repeated this process for the other three strengthening exericses. We caluculated overall AUC (Cell D, Row 4) by adding together the four integral AUC values. To calculate the overall cumulative dose prescribed by the therapist and completed by the participant across the five face-to-face exercise sessions, we added together the overall AUC values (i.e. 150+130+120+120=520). We repeated this process for both hands to take into account any differences in dose between left and right sides. In participant 5’s case, the dose used for both hands was identical, therefore the overall dose of strengthening exercise prescribed by the therapist and completed by the participant was 1040.

**Table 4** Calculating integral AUC and overall AUC in Microsoft Excel

| Row | Column | | | | | | | | | | | | |
| --- | --- | --- | --- | --- | --- | --- | --- | --- | --- | --- | --- | --- | --- |
|  | Cell A | Cell B | Cell C | Cell D | Cell E | Cell F | Cell G | Cell H | Cell I | Cell J | Cell K | Cell L | Cell M |
|  | Exercise  session | Eccentric wrist extension | | | Gross grip | | | Finger adduction | | | Finger pinch | | |
|  |  | Dose | Integral  AUC | Overall  AUC | Dose | Integral  AUC | Overall  AUC | Dose | Integral  AUC | Overall  AUC | Dose | Integral  AUC | Overall  AUC |
| Row 1 | 1 | 50 | 50 |  | 40 | 40 |  | 40 | 40 |  | 40 | 40 |  |
| Row 2 | 2 | 50 | 50 |  | 40 | 45 |  | 40 | 40 |  | 40 | 40 |  |
| Row 3 | 3 | 50 | 25 |  | 50 | 25 |  | 40 | 20 |  | 40 | 20 |  |
| Row 4 | 4 | 0 | 25 | 150 | 0 | 20 | 130 | 0 | 20 | 120 | 0 | 20 | 120 |
| Row 5 | 5 | 50 |  |  | 40 |  |  | 40 |  |  | 40 |  |  |

**REFERENCES:**

Boniface, G., Gandhi, V., Norris, M., Williamson, E., Kirtley, S., & O’Connell, N. E. (2020). A systematic review exploring the evidence reported to underpin exercise dose in clinical trials of rheumatoid arthritis. *Rheumatology*. doi:10.1093/rheumatology/keaa150

Matthews, J. N., Altman, D. G., Campbell, M. J., & Royston, P. (1990). Analysis of serial measurements in medical research. *300*(0959-8138 (Print)). doi:<https://doi.org/10.1136/bmj.300.6719.230>

Pruessner, J. C., Kirschbaum, C., Meinlschmid, G., & Hellhammer, D. H. (2003). Two formulas for computation of the area under the curve represent measures of total hormone concentration versus time-dependent change. *Psychoneuroendocrinology, 28*(7), 916-931. doi:0.1016/s0306-4530(02)00108-7

Slade, S. C., Dionne, C. E., Underwood, M., & Buchbinder, R. (2016). Consensus on Exercise Reporting Template (CERT): explanation and elaboration statement. *British Journal of Sports Medicine, 50*(23), 1428-1437. doi:10.1136/bjsports-2016-096651

Slade, S. C., Dionne, C. E., Underwood, M., Buchbinder, R., Beck, B., Bennell, K., . . . Cup, E. (2016). Consensus on exercise reporting template (CERT): modified Delphi study. *Physical therapy, 96*(10), 1514-1524. doi:10.2522/ptj.20150668
