# Supplementary material for: Understanding prescribed dose in hand strengthening exercise for rheumatoid arthritis: A secondary analysis of the SARAH trial
Source: Musculoskeletal Care. 2022 May 16;20(4):899–907. doi: 10.1002/msc.1646 (PMC10084296; doi:10.1002/msc.1646)
Supplement: Supplementary file 2 — Supplementary Material [file MSC-20-899-s002.docx]

**APPENDIX B**

**Table 1.** Characteristics of non-hand specific study sample variables (N=246)

| **Indicator variable** | **Participants with dose** | **Participants with no dose** | **Participants with missing dose** |
| --- | --- | --- | --- |
|  |  |  |  |
|  | **n=222 (444 hands)** | **n=19 (38 hands)** | **n=5 (10 hands)** |
|  |  |  |  |
| ***Participant age, mean (SD):*** | 61.6 (11.9) | 56.4 (15.5) | 62.4 (13.0) |
|  |  |  |  |
| ***Participant age, n (%):*** |  |  |  |
| Less than 45 years | 22.0 (9.9) | 6.0 (31.6) | 0.0 (0.0) |
| 45-54 years | 35.0 (15.8) | 3.0 (15.8) | 2.0 (40.0) |
| 55-64 years | 73.0 (32.9) | 2.0 (10.5) | 1.0 (20.0) |
| 65 and over | 92.0 (41.4) | 8.0 (42.1) | 2.0 (40.0) |
| Missing data | 0.0 (0.0) | 0.0 (0.0) | 0.0 (0.0) |
|  |  |  |  |
| ***Sex, n (%):*** |  |  |  |
| Male | 54.0 (24.3) | 3.0 (15.8) | 1.0 (20.0) |
| Female | 168.0 (75.7) | 16.0 (84.2) | 4.0 (80.0) |
| Missing data | 0.0 (0.0) | 0.0 (0.0) | 0.0 (0.0) |
|  |  |  |  |
| ***Years diagnosed with RA, mean (SD):*** | 12.4 (10.1) | 14.0 (10.3) | 5.8 (3.8) |
| Missing data | 1.0(0.5) | 0.0 (0.0) | 0.0 (0.0) |
|  |  |  |  |
| ***Type of therapist treating, n (%):*** |  |  |  |
| Physiotherapist | 79.0 (35.6) | 0.0 (0.0) | 1.0 (20.0) |
| Occupational therapist | 143.0 (64.4) | 4.0 (21.1) | 4.0 (80.0) |
| Missing data | 0.0 (0.0) | 15.0 (78.9) | 0.0 (0.0) |
|  |  |  |  |
| ***Treating therapist grade, n (%):*** |  |  |  |
| Job band 5 | 10.0 (4.5) | 1.0 (5.3) | 0.0 (0.0) |
| Job band 6 | 133.0 (59.9) | 3.0 (15.8) | 2.0 (40.0) |
| Job band 7 | 70.0 (31.5) | 0.0 (0.0) | 2.0 (40.0) |
| Missing data | 9.0 (4.1) | 15.0 (78.9) | 1.0 (20.0) |
|  |  |  |  |
| ***Pain frequency, n (%):*** |  |  |  |
| Always/often | 137.0 (61.7) | 14.0 (73.7) | 3.0 (60.0) |
| Sometimes | 59.0 (26.6) | 4.0 (21.1) | 2.0 (40.0) |
| Rarely/never | 26.0 (11.7) | 1.0 (5.3) | 0.0 (0.0) |
| Missing data | 0.0 (0.0) | 0.0 (0.0) | 0.0 (0.0) |
|  |  |  |  |
| ***Pain severity, n (%):*** |  |  |  |
| Very mild/mild | 55.0 (24.8) | 4.0 (21.1) | 1.0 (20.0) |
| Moderate | 116.0 (52.3) | 7.0 (36.8) | 4.0 (80.0) |
| Severe/very severe | 45 (20.3) | 8.0 (42.1) | 0.0 (0.0) |
| Missing data | 6.0 (2.7) | 0.0 (0.0) | 0.0 (0.0) |
|  |  |  |  |
| ***Accomplished less than liked (SF-12), n (%):*** |  |  |  |
| All/most of the time | 38.0 (17.1) | 5.0 (26.3) | 1.0 (20.0) |
| Some of the time | 52.0 (23.4) | 5.0 (26.3) | 2.0 (40.0) |
| A little/none of the time | 132.0 (59.5) | 9.0 (47.4) | 2.0 (40.0) |
| Missing data | 0.0 (0.0) | 0.0 (0.0) | 0.0 (0.0) |
|  |  |  |  |
| ***Feeling downhearted or low (SF-12), n (%):*** |  |  |  |
| All/Most of the time | 17.0 (7.7) | 4.0 (21.1) | 1.0 (20.0) |
| Some of the time | 52.0 (23.4) | 4.0 (21.1) | 2.0 (40.0) |
| A little/none of the time | 140.0 (63.1) | 11.0 (57.9) | 2.0 (40.0) |
| Missing data | 0.0 (0.0) | 0.0 (0.0) | 0.0 (0.0) |
|  |  |  |  |
| ***Confidence to perform exercise, median (IQR):*** | 7.0 (5.0, 9.0) | 6.0 (3.0, 8.0) | 6.0 (4.5, 9.2) |
| Missing data, n (%) | 1.0 (0.5) | 0.0 (0.0) | 0.0 (0.0) |
|  |  |  |  |
| ***Hand/wrist swollen joint count, median (IQR):*** | 2.0 (1.0, 7.0) | 3.0 (2.0, 7.0) | 1.0 (0.0, 3.2) |
| Missing data, n (%) | 0.0 (0.0) | 0.0 (0.0) | 0.0 (0.0) |
|  |  |  |  |
| ***Hand/wrist joint tenderness count, median (IQR):*** | 3.0 (1.0, 8.0) | 5.0 (1.0, 9.0) | 2.0 (2.0. 3.7) |
| Missing data, n (%) | 0.0 (0.0) | 0.0 (0.0) | 0.0 (0.0) |

**Table 2**. Characteristics of hand specific study sample variables (N=246)

|  | **Participants with dose** | | **Participants with no dose** | | **Participants with missing dose** | |
| --- | --- | --- | --- | --- | --- | --- |
|  | **n=222 (444 hands)** | | **n=19 (38 hands)** | | **n=5 (10 hands)** | |
| **Indicator variable** | **Left hand** | **Right hand** | **Left hand** | **Right hand** | **Left hand** | **Right hand** |
|  |  |  |  |  |  |  |
| **Metacarpophalangeal joint deformity**, (either radial/ulnar side), n (%): | 186.0 (83.8) | 182.0 (82.0) | 16.0 (84.2) | 13.0 (68.4) | 3.0 (60.0) | 3.0 (60.0) |
| No deformity, n (%): | 35.0 (15.8) | 39.0 (17.6) | 3.0 (15.8) | 6.0 (31.6) | 2.0 (40.0) | 2.0 (40.0) |
| Missing data, n (%): | 1.0 (0.5) | 1.0 (0.5) | 0.0 (0.0) | 0.0 (0.0) | 0.0 (0.0) | 0.0 (0.0) |
|  |  |  |  |  |  |  |
| **Active wrist extension, median (IQR):** | 50.0 (38.7, 60.5) | 50.0 (35.5, 58.5) | 54.0 (28.0, 62.0) | 50.0 (31.0, 60.0) | 52.0 (34.5, 56.0) | 48.0 (33.0, 55.0) |
| Missing data, n (%): | 0.0 (0.0) | 0.0 (0.0) | 1.0 (5.3) | 1.0 (5.3) | 0.0 (0.0) | 0.0 (0.0) |
|  |  |  |  |  |  |  |
| **Thumb opposition, median (IQR):** | 9.0 (8.0, 10.0) | 9.0 (7.0, 9.0) | 9.0 (7.0, 9.0) | 9.0 (6.0, 9.0) | 9.0 (9.0, 9.5) | 9.0 (9.0, 10.0) |
| Missing data, n (%): | 0.0 (0.0) | 0.0 (0.0) | 0.0 (0.0) | 0.0 (0.0) | 0.0 (0.0) | 0.0 (0.0) |
|  |  |  |  |  |  |  |
| **^†^Combined finger flexion, median (IQR):** | 5.0 (0.0, 24.0) | 5.5 (0.0, 21.5) | 3.0 (0.0, 21.0) | 6.5 (0.0, 20.5) | 0.0 (0.0, 13.7) | 0.0 (0.0, 15.5) |
| Missing data, n (%): | 0.0 (0.0) | 1.0 (0.5) | 0.0 (0.0) | 0.0 (0.0) | 0.0 (0.0) | 0.0 (0.0) |
|  |  |  |  |  |  |  |
| **^‡^Gross grip strength, median (IQR):** | 106.0 (64.5, 150.5) | 111.0 (63.5, 163.0) | 72.0 (52.0, 120.0) | 102.0 (50.0, 131.0) | 126.0 (105.0, 285.5) | 142.0 (107.5, 256.0) |
| Missing data, n (%): | 1.0 (0.5) | 1.0 (0.5) | 0.0 (0.0) | 0.0 (0.0) | 0.0 (0.0) | 0.0 (0.0) |
|  |  |  |  |  |  |  |
| **^‡^Pinch grip strength, median (IQR):** | 33.0 (21.0, 46.5) | 33.0 (21.0, 50.0) | 26.0 (17.0, 35.0) | 31.0 (22.0, 42.0) | 33.0 (27.5, 71.5) | 36.0 (32.0, 78.5) |
| Missing data, n (%): | 5.0 (2.3) | 3.0 (1.4) | 0.0 (0.0) | 0.0 (0.0) | 0.0 (0.0) | 0.0 (0.0) |
|  |  |  |  |  |  |  |
| **Overall hand function (MHQ sub-scale), median (IQR):** | 50.0 (40.0, 65.0) | 50.0 (40.0, 65.0) | 45.0 (40.0, 55.0) | 45.0 (40.0, 50.0) | 65.0 (50.0, 70.0) | 65.0 (50.0, 72.5) |
| Missing data, n (%): | 0.0 (0.0) | 0.0 (0.0) | 0.0 (0.0) | 0.0 (0.0) | 0.0 (0.0) | 0.0 (0.0) |

^†^ Mean result of index, middle, ring and little fingers, ^‡^ Mean result of three test attempts
